# Predicting all-cause and lung cancer mortality using emphysema score progression rate between baseline and follow-up chest CT images: A comparison of risk model performances

**DOI:** 10.1371/journal.pone.0212756

**Published:** 2019-02-21

**Authors:** Anton Schreuder, Colin Jacobs, Leticia Gallardo-Estrella, Mathias Prokop, Cornelia M. Schaefer-Prokop, Bram van Ginneken

**Affiliations:** 1 Diagnostic Image Analysis Group, Department of Radiology and Nuclear Medicine, Radboudumc, Nijmegen, the Netherlands; 2 Thirona, Nijmegen, the Netherlands; 3 Department of Radiology, Meander Medisch Centrum, Amersfoort, the Netherlands; 4 Fraunhofer MEVIS, Bremen, Germany; University of Central Florida (UCF), UNITED STATES

## Abstract

**Purpose:**

Normalized emphysema score is a protocol-robust CT biomarker of mortality. We aimed to improve mortality prediction by including the emphysema score progression rate–its change over time–into the models.

**Method and materials:**

CT scans from 6000 National Lung Screening Trial CT arm participants were included. Of these, 1810 died (445 lung cancer-specific). The remaining 4190 survivors were sampled with replacement up to 24432 to approximate the full cohort. Three overlapping subcohorts were formed which required participants to have images from specific screening rounds. Emphysema scores were obtained after resampling, normalization, and bullae cluster analysis of the original images. Base models contained solely the latest emphysema score. Progression models included emphysema score progression rate. Models were adjusted by including baseline age, sex, BMI, smoking status, smoking intensity, smoking duration, and previous COPD diagnosis. Cox proportional hazard models predicting all-cause and lung cancer mortality were compared by calculating the area under the curve per year follow-up.

**Results:**

In the subcohort of participants with baseline and first annual follow-up scans, the analysis was performed on 4940 participants (23227 after resampling). Area under the curve for all-cause mortality predictions of the base and progression models 6 years after baseline were 0.564 (0.564 to 0.565) and 0.569 (0.568 to 0.569) when unadjusted, and 0.704 (0.703 to 0.704) to 0.705 (0.704 to 0.705) when adjusted. The respective performances predicting lung cancer mortality were 0.638 (0.637 to 0.639) and 0.643 (0.642 to 0.644) when unadjusted, and 0.724 (0.723 to 0.725) and 0.725 (0.725 to 0.726) when adjusted.

**Conclusion:**

Including emphysema score progression rate into risk models shows no clinically relevant improvement in mortality risk prediction. This is because scan normalization does not adjust for an overall change in lung density. Adjusting for changes in smoking behavior is likely required to make this a clinically useful measure of emphysema progression.

## Introduction

Visual assessment of emphysema extent in CT images of the lungs is a time-consuming process that requires training and is prone to subjectivity [1]. To solve these issues, a quantitative measure of emphysema was introduced [2]. Emphysema score (ES) is defined as the percentage voxels below -950 HU in the lungs [3, 4]. However, its value as a clinical risk predictor is limited due to its dependence on different imaging protocols and reconstruction algorithms [5, 6]; ES from different centers cannot be compared. Recently, normalized ES was introduced to overcome this issue by applying a normalization algorithm before calculating the ES [7]. It was subsequently shown using images from the National Lung Screening Trial (NLST) CT cohort that mortality risk groups based on normalized ES could better distinguish high- from low-risk individuals compared to those based on the original ES [8, 9]. From this point on in this paper, “normalized ES” is referred to as “ES” unless stated otherwise.

Having shown that ES is a protocol-robust CT biomarker, we wanted to investigate whether mortality predictions would improve if a previous CT image was available, enabling the simple calculation of ES change per year (ES progression rate, EPR). This would be especially relevant in a lung cancer CT screening setting where a large, high-risk group is scanned on a yearly basis; even small improvements in a risk model’s accuracy can have affect many people. Compared to having measurements at only one time point, disease prognosis should become more accurate when the disease progression rate is known. We hereby hypothesized that EPR could be used as an additional variable to improve the prediction accuracy of all-cause mortality and lung cancer mortality. In the situation where more than one prior CT image is available, it was assumed that a larger absolute difference in ES resulting from more time between the scans would decrease the proportion associated with within-participant random measurement errors. Our second hypothesis was that measuring EPR between scans separated by a larger time interval would improve the risk prediction accuracy.

## Materials and methods

### Scans and data

Permission to use anonymized scans and data from the NLST study was obtained by the National Cancer Institute Cancer Data Access System under project ID NLST-422. In summary, the NLST was a multicenter randomized controlled trial which enrolled 53454 heavy smokers in the USA with the aim to investigate whether annual low-dose chest CT screening significantly reduces the lung cancer mortality compared to chest radiography [8]. The trial took place between 2002 and 2010; participants were screened in three annual rounds (T0, T1, and T2) and followed for another five years for outcome measures. In the CT arm, 26309, 24715, and 24102 participants were screened in T0, T1, and T2, respectively; 1810 participants with at least one CT image available were confirmed dead, of which 445 died specifically of lung cancer (if not endpoint verification certified, is stated as cause of death on the death certificate). Note that published results may differ slightly from those in the database due to updates and corrections, and that incomplete or corrupted images were excluded from the study.

The NLST also kept track of the time between each screening round and the event, enabling survival analysis to be performed (see “Statistical analysis” section). Seven patient characteristics as collected from baseline questionnaires were selected to be included in the prediction model as adjustment factors to test whether ES and EPR are independent risk factors. These known predictors of emphysema were: age (years), sex, BMI (five categories: <18.5, 18.5–25, 25–30, 30–35, and ≥35), smoking status, cigarette smoking intensity (pack years), cigarette smoking duration (years), and previous diagnosis of COPD.

### Data selection and subcohorts

The same 6000 participants used by Gallardo-Estrella and colleagues [9] were considered, which consisted of all deceased participants and a random selection of non-deceased participants from the NLST CT arm. Three overlapping study subcohorts were formed based on which screening round images per participant were available: the T0-T1, T0-T2, and T0-T1-T2 subcohorts. The T0-T1 and T0-T2 subcohorts were used to test the first hypothesis: that EPR is of added value for mortality risk prediction regardless of whether EPR was calculated between T0 and T1 or between T0 and T2 scans. The T0-T1-T2 subcohort excluded the largest number of available participants and therefore had a lower statistical power than the prior two cohorts, but a common data set was required to formally test the second hypothesis: that a two-year EPR would improve the prediction accuracy compared to a one-year EPR.

### Emphysema quantification

ES was calculated using CIRRUS Lung Quantification (Diagnostic Image Analysis Group; Fraunhofer MEVIS). This process starts with the segmentation of the lungs using an algorithm based on region growing and morphological smoothing [10]. Subsequently, the images are resampled to 3mm slice thickness, normalized [7], and bullae analysis [11] is performed. The percentage of lung voxels less than -950 HU is calculated from the final image as ES [7, 12]. EPR was defined as the difference between the latest available ES and the ES from the prior scan, adjusted by the number of years between the two scans, given by the following equation:

$$EPR=\frac{{ES}_{latest}–{ES}_{prior}}{days\;in\;between\;scans}\times 365.25$$

### Statistical analysis

Data was collected and organized in Microsoft Excel; statistical analysis was performed using R statistical analysis package V.3.4.3. As we did not have the complete NLST non-deceased CT cohort, this subcohort was resampled to simulate the full NLST CT cohort (n = 24432) before excluding participants to form this study’s three subcohorts (see “Data selection and subcohorts”).

Cox proportional hazards regression analysis was performed (R package “survival”) to predict the risk of two outcomes: all-cause mortality and lung cancer mortality. For each subcohort, a base model and a progression model were created; two progression models were created using the T0-T1-T2 subcohort. The base model contained one variable: the latest ES (at T1 or T2). The progression models additionally included EPR (average ES change per year between T0 and T1, T0 and T2, or T1 and T2).

The analysis was subsequently performed on the adjusted version of the above-mentioned models, which included seven clinical patient characteristics (see section “Scans and data”). In total, 28 models were created; the ES and EPR beta coefficients and hazard ratios including 95% confidence intervals and Wald statistic p-values were recorded for each model. Beta coefficients represent the change in the natural log of the hazard ratio for each unit change in the associated risk factor; the estimated hazard ratio is calculated by multiplying each risk factor with its beta coefficient, summing them, and solving the exponential function of that value. Kaplan-Meier curves were computed with 95% confidence intervals using R package “ggfortify” to visualize the ability of each model to distinguish between different risk group percentiles of participants. The same risk group percentiles were used as Gallardo-Estrella et al. [9] (percentiles 0 to 60, 60 to 80, and 80 to 100), but were further subdivided into more groups of no smaller than 10% of the study population when there was a good visual separation.

The proportional hazards assumption was tested using Schoenfeld residuals (R package “survival”); a p-value below 0.05 is interpretable as a violation of this assumption. Martingale residual plots were used to visually assess whether each continuous variable had a linear relationship with the log hazard (R package “survminer”). The likelihood ratio test, Wald test, and logrank test p-values were obtained for each Cox model (R package “survival”) to test goodness of fit.

To compare the predictive accuracy of each model, two time-dependent statistical methods were applied at each year follow-up. Area under the receiver operating characteristic curve (AUC) was used with the support of R package “ROCR” [13]; estimates were from applying the models to the bootstrap data. A value of 0.5 indicates that the model has no predictive ability. When comparing the AUCs of two models, a value closer to 1 indicates a higher accuracy (granted that the 95% confidence intervals do not encompass the other model’s AUC). Average continuous net reclassification improvement (NRI)–defined as “1/2 NRI(>0)” by Pencina and colleagues [14]–was calculated using R package “survIDINRI” [15, 16]. To calculate NRI, the net percentages of subjects with and without the event of interest correctly reclassified with a higher and lower risk score, respectively, are summed and halved (maximum range: -100% to 100%); positive scores indicate that the new model is more accurate (granted that the 95% confidence intervals do not encompass 0). 95% confidence intervals were obtained from 1000 bootstrap iterations with replacement for both methods.

To simulate the models’ application in a screening setting, we calculated the sensitivity, specificity, positive predictive value, negative predictive value, and the ratio of non-deceased to deceased at several pragmatic percentile cut-off points. 95% confidence intervals were calculating using the Wilson procedure with continuity correction [17].

## Results

### Study participants

Compared to non-deceased participants, more of the deceased participants were older, male, had a higher BMI, were active smokers with a more extensive smoking history, and were diagnosed with COPD (Table 1). Regarding adjustment factors, 17 participants were missing values for the variable “BMI” and 14 were missing “previous diagnosis of COPD”; these participants were excluded from the analysis.

**Table 1:** Baseline characteristics of participants with a baseline scan.

Characteristic	Deceased (n = 1737)	Non-deceased (n = 4003)	P-value^a^	Lung cancer death (n = 428)	Non-lung cancer death (n = 5312)	P-value^a^
**T1 ES (IQR)^b^**	0.52 (0.11 to 2.43)	0.31 (0.07 to 1.33)	< .001	1.04 (0.20 to 3.72)	0.33 (0.08 to 1.49)	< .001
**T2 ES (IQR)^c^**	0.65 (0.12 to 3.17)	0.35 (0.08 to 1.49)	< .001	1.40 (0.26 to 4.83)	0.38 (0.09 to 1.67)	< .001
**T0-T1 EPR (IQR) ^b^**	0.03 (-0.10 to 0.41)	0.02 (-0.06 to 0.25)	.456	0.08 (-0.10 to 0.58)	0.02 (-0.06 to 0.26)	.022
**T0-T2 EPR (IQR) ^c^**	0.06 (-0.07 to 0.73)	0.04 (-0.03 to 0.37)	.102	0.19 (-0.09 to 1.14)	0.04 (-0.04 to 0.41)	.002
**Median age, years (IQR)**	63 (59 to 68)	60 (57 to 64)	< .001	64 (59 to 68)	61 (57 to 65)	< .001
**No. Female (%)**	521 (30.0)	1653 (41.3)	< .001	145 (33.9)	2029 (38.2)	.086
**Median BMI, kg/m^2^ (IQR)**	27.0 (24.1 to 30.7)	27.1 (24.5 to 30.7)	.032	26.3 (24.0 to 29.3)	27.2 (24.4 to 30.7)	< .001
**No. active smokers (%)**	1032 (59.4)	1863 (46.5)	< .001	266 (62.1)	2629 (49.5)	< .001
**Median smoking intensity, pack-years (IQR)**	56 (44 to 78)	47 (39 to 66)	< .001	59 (46 to 84)	50 (40 to 68)	< .001
**Median smoking duration, years (IQR)**	43 (39 to 49)	40 (35 to 44)	< .001	44 (40 to 49)	40 (36 to 45)	< .001
**No. previous COPD diagnoses (%)**	178 (10.3)	181 (4.5)	< .001	47 (11.0)	312 (5.9)	< .001

BMI, body mass index; IQR, interquartile range; ES, emphysema score; T0, baseline screening round; T1, first annual follow-up screening round; T2, second annual follow-up screening round.

^a^ Mann-Whitney U test for nonparametric continuous variables; Pearson χ^2^ test for binary variables.

^b^ Of all participants with both T0 and T1 scans (T0-T1 subcohort).

^c^ Of all participants with both T0 and T2 scans (T0-T2 subcohort).

**Fig 1:**
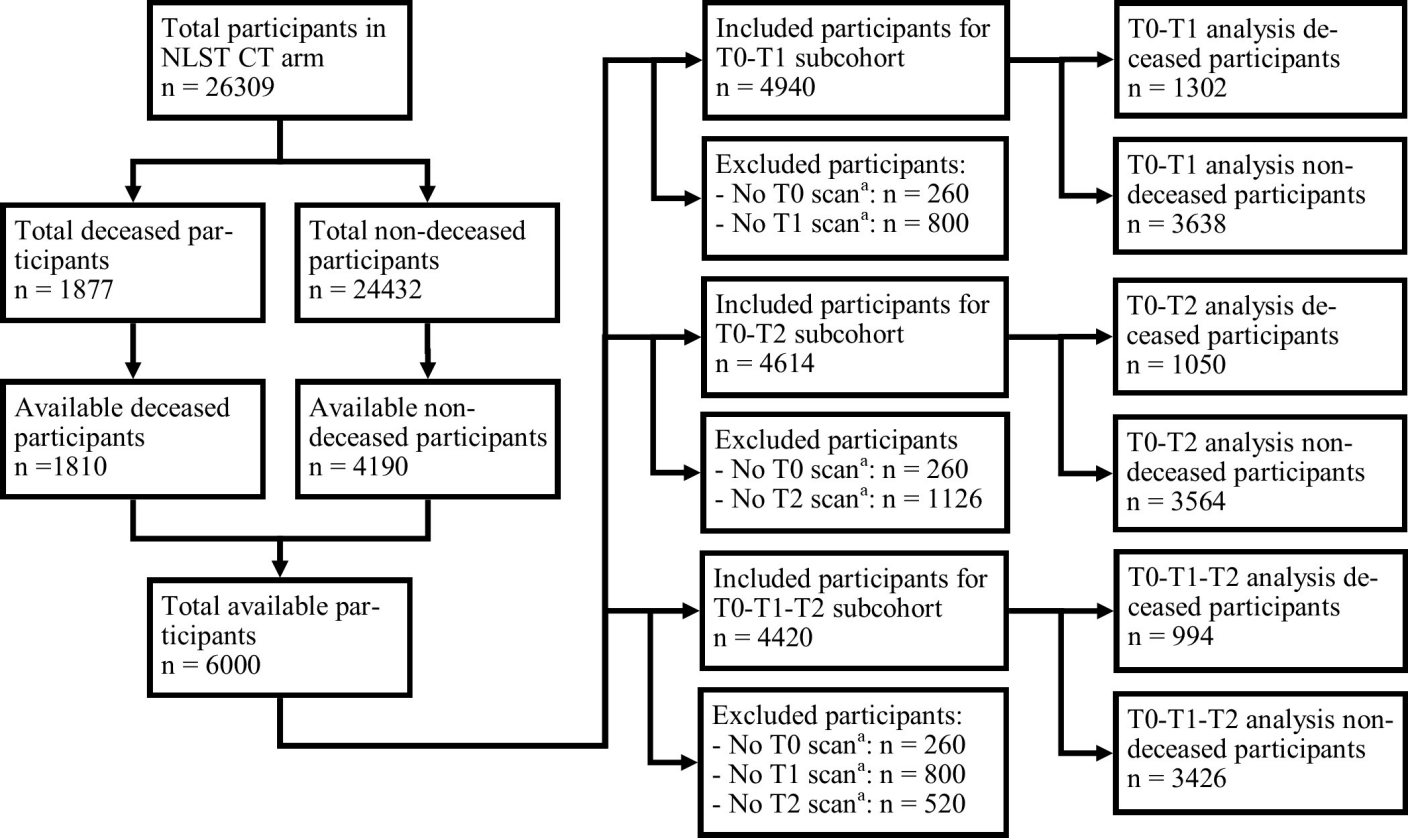
Participant selection flow chart. NLST, National Lung Screening Trial; T0, baseline screening round; T1, first annual follow-up screening round; T2, second annual follow-up screening round. ^a^ CT images may be missing due to the participant skipping a screening round, corrupted images, or incomplete images. Each subsequent exclusion count does not overlap with the previous criteria.

Out of the 6000 available participants, 4940 (82.3%) were included in the T0-T1 subcohort, 4614 (76.9%) were included in the T0-T2 subcohort, and 4420 (73.7%) were included in the T0-T1-T2 subcohort (Fig 1). The respective percentage of deaths per subcohort were 26.4% (1302/4940), 22.8% (1050/4614), and 22.5% (994/4420); 5.7% (281/4940), 4.7% (215/4614), and 4.6% (202/4420) lung cancer-specific deaths, respectively. The non-deceased participants were first resampled to the full cohort equivalent of 24432 non-deceased participants, then excluded based on each subcohort’s criteria. With both deceased and resampled non-deceased, the final subcohort sizes used for regression analysis were 23227, 22592, and 21679 participants, respectively.

**Fig 2:**
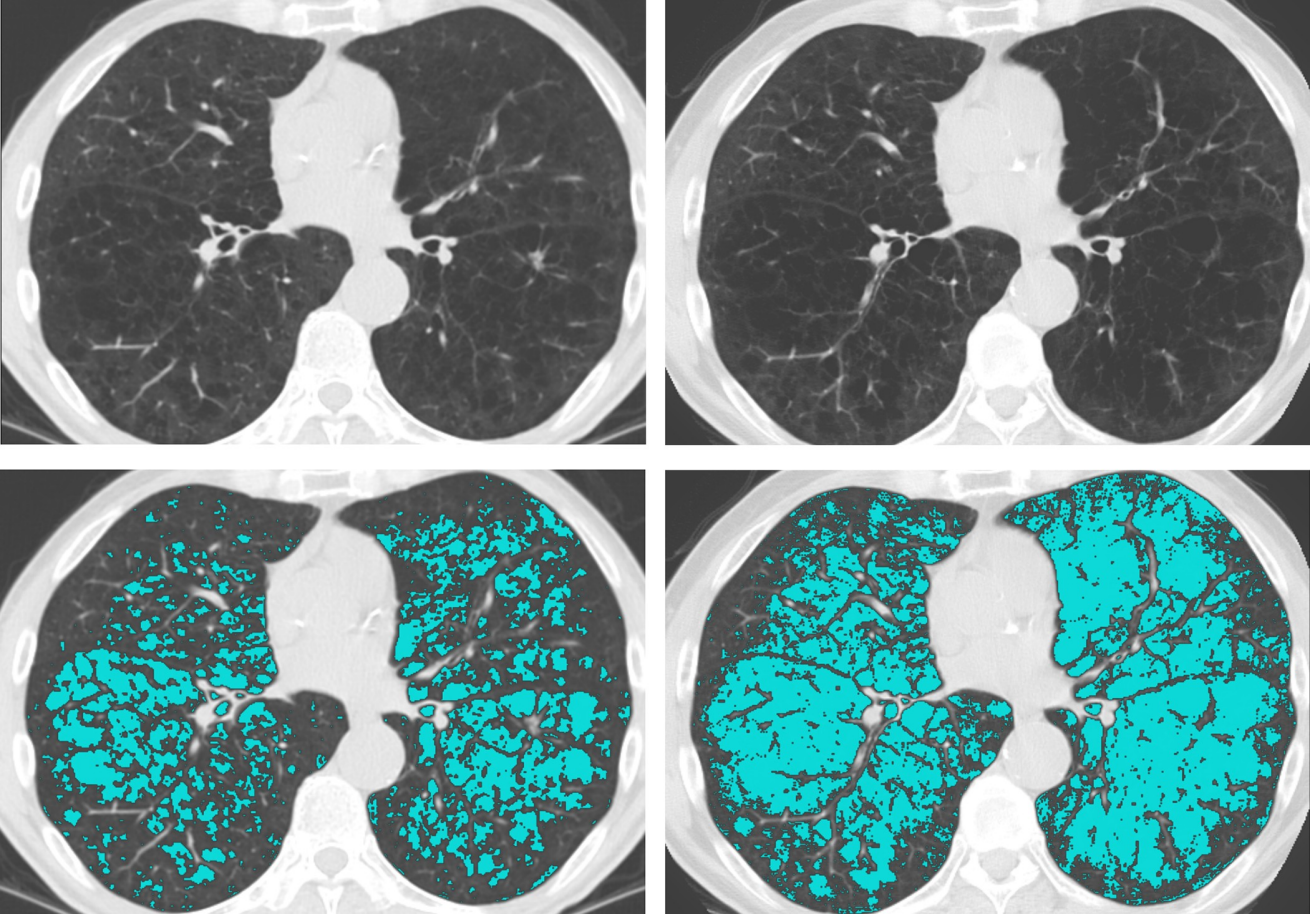
Screenshots of a positive emphysema score progression case. Four screenshots with emphysema score overlays from one participant are displayed. The left-side screenshots are from the baseline scan; the right-side screenshots are from the first annual follow-up scan. The top screenshots are without the emphysema score overlay. This participant had an emphysema score progression rate of 21.6.

**Fig 3:**
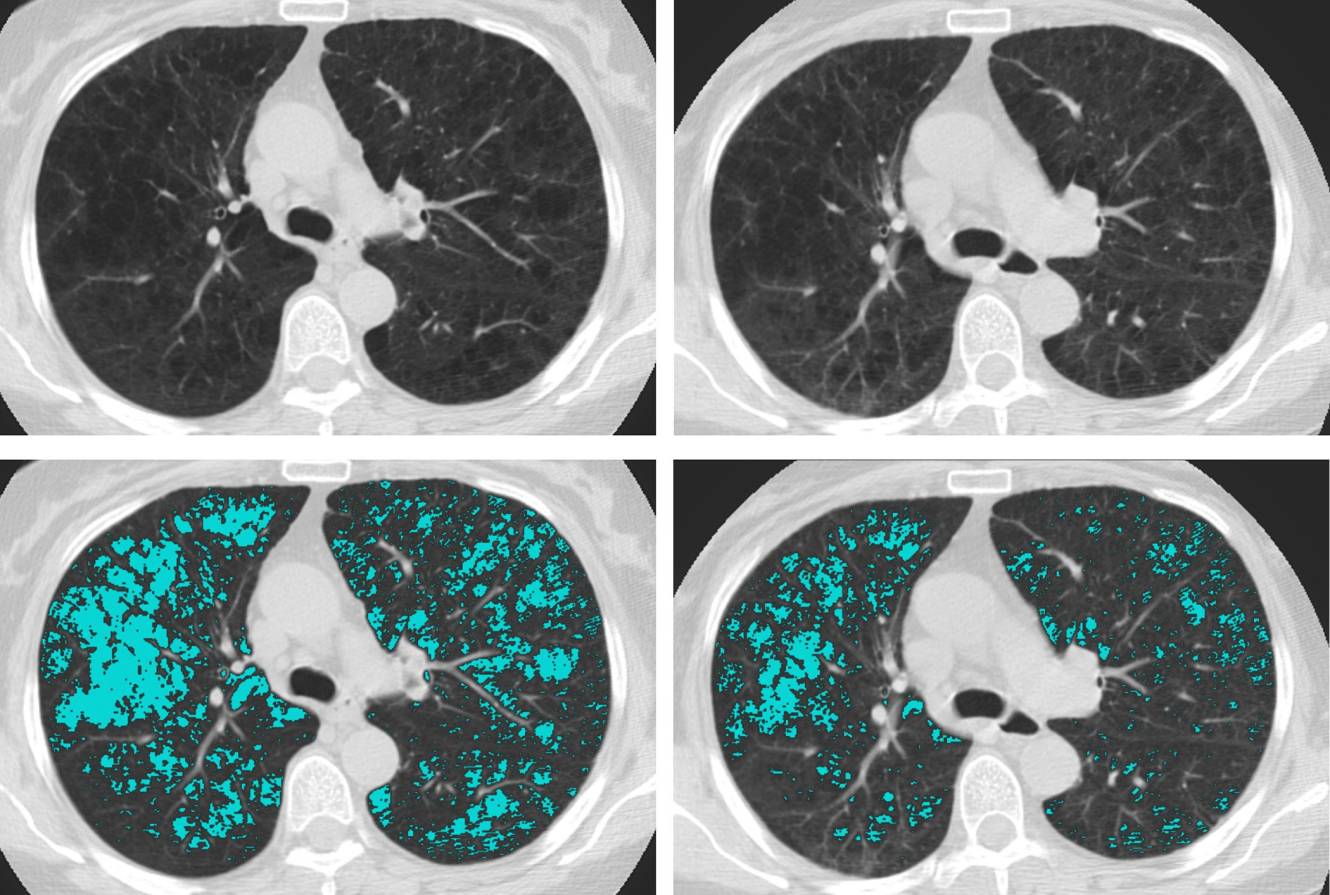
Screenshots of a negative emphysema score progression case. Four screenshots with emphysema score overlays from one participant are displayed. The left-side screenshots are from the baseline scan; the right-side screenshots are from the first annual follow-up scan. The top screenshots are without the emphysema score overlay. This participant had an emphysema score progression rate of -10.3.

Among all participants with a baseline scan, the derived ESs for each screening round as well as the EPRs between T0 and T1 and T0 and T2 can be found in the supplementary dataset (S1 Dataset). Figs 2 and 3 show the normalized T0 and T1 CT images with and without ES overlays of two participants, the former with an EPR of 21.6 and the latter -10.3.

### Cox model assumptions

All variables in all models had a p-value greater than 0.05 when testing for the proportional hazard assumption. All models had p<0.0001 for the goodness of fit tests (likelihood ratio test, Wald test, and logrank test). The distribution of ES was right skewed, but attempts to transform this variable (i.e., log transformation, absolute value transformation, square root transformation, and polynomial transformation) did not maintain linearity. Similarly, transforming EPR had little effect due to the small spread. The other continuous adjustment variables (age, pack years, and smoking duration) remained slightly nonlinear regardless of transformation and were ultimately also left untransformed.

### Base versus progression models

**Fig 4:**
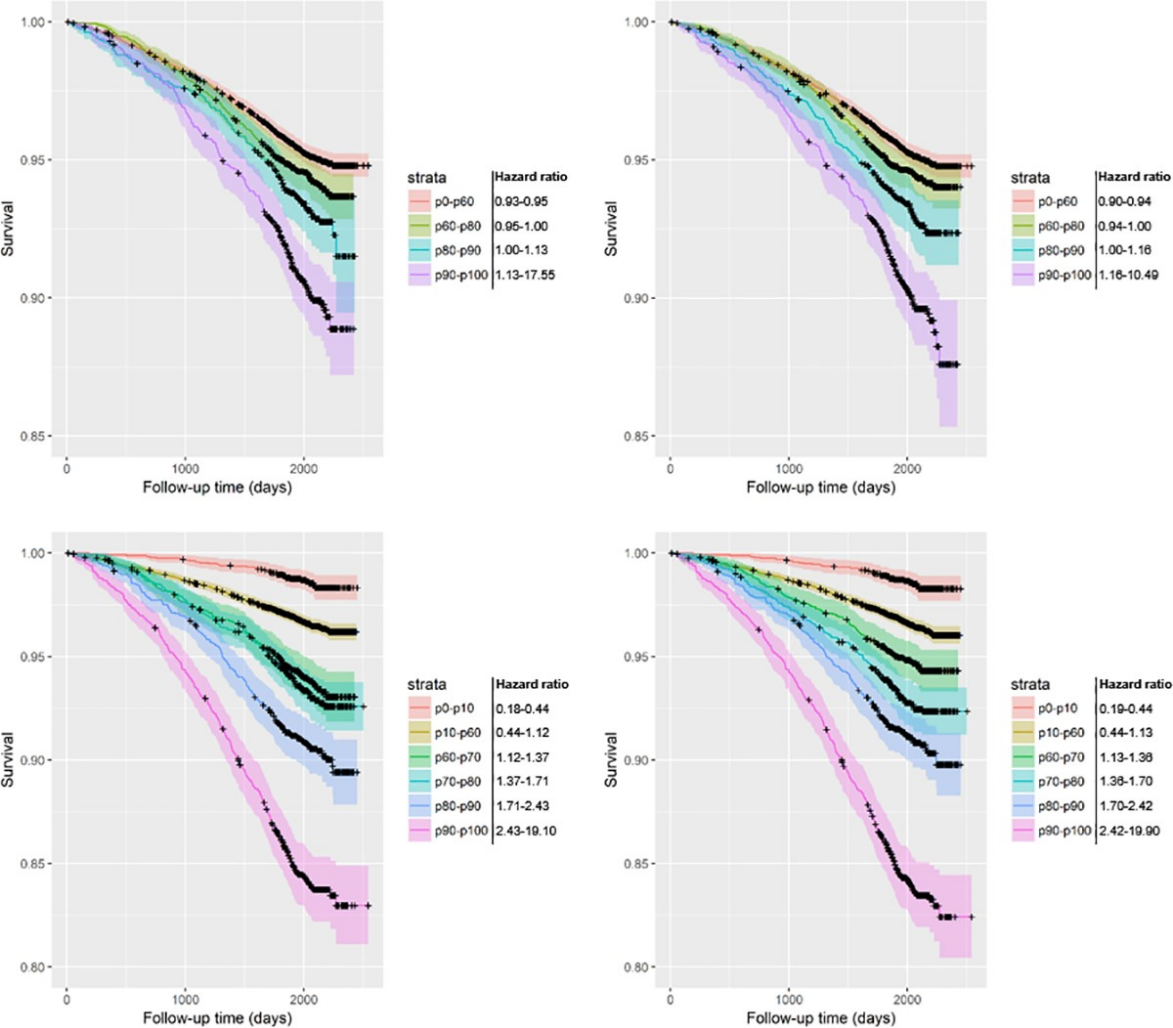
Kaplan-Meier curves of all-cause mortality survival predictions including 95% confidence intervals (resampled T0-T1 subcohort). The base models only contain the variable emphysema score; the progression models include both emphysema score and average change of emphysema score per year. Risk groups are divided by risk percentiles and their corresponding hazard ratios as displayed in the key. The resampled T0-T1 subcohort contains participants with both T0 and T1 scans resampled to the original cohort size. Upper-left: unadjusted base model; Upper-right: unadjusted one-year progression model; Lower-left: adjusted base model; Lower-right: adjusted one-year progression model.

Table 2 displays the beta coefficients and hazard ratios of the ES and EPR for all models developed from the resampled T0-T1 and T0-T2 subcohorts. ES was a statistically significant variable in all models (p<0.001); this also applied to EPR in the all-cause mortality prediction models. EPR was less accurate for lung cancer mortality in the unadjusted models, and not of added benefit when adjusting for patient characteristics (p>0.05). Beta coefficients and hazard ratios of the adjustment factors are displayed in Tables 3 and 4. The model performances are visualized as Kaplan-Meier curves in Figs 4 through 7.

**Fig 5:**
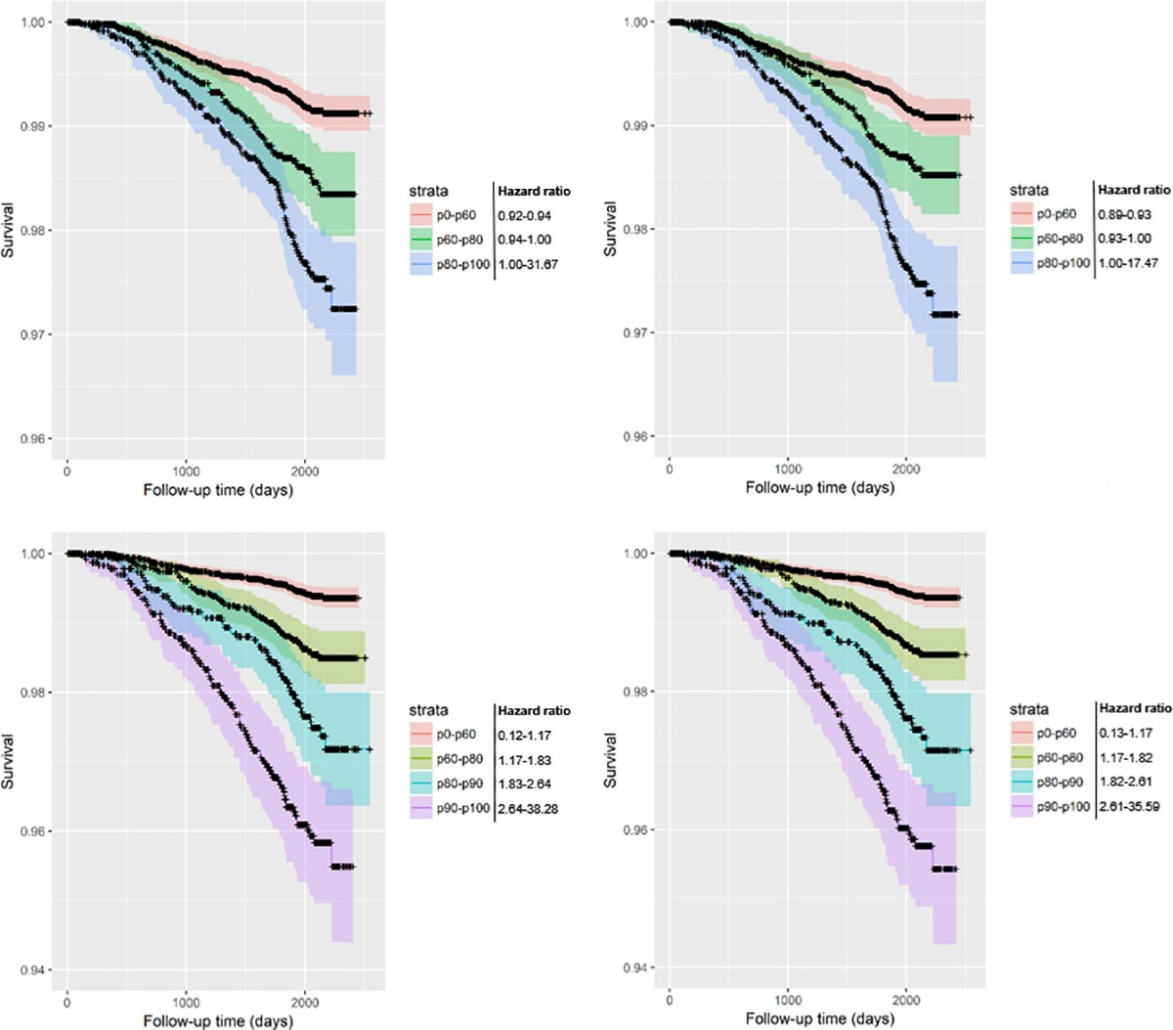
Kaplan-Meier curves of lung cancer mortality survival predictions including 95% confidence intervals (resampled T0-T1 subcohort). The base models only contain the variable emphysema score; the progression models include both emphysema score and average change of emphysema score per year. Risk groups are divided by risk percentiles and their corresponding hazard ratios as displayed in the key. The resampled T0-T1 subcohort contains participants with both T0 and T1 scans resampled to the original cohort size. Upper-left: unadjusted base model; Upper-right: unadjusted one-year progression model; Lower-left: adjusted base model; Lower-right: adjusted one-year progression model.

**Fig 6:**
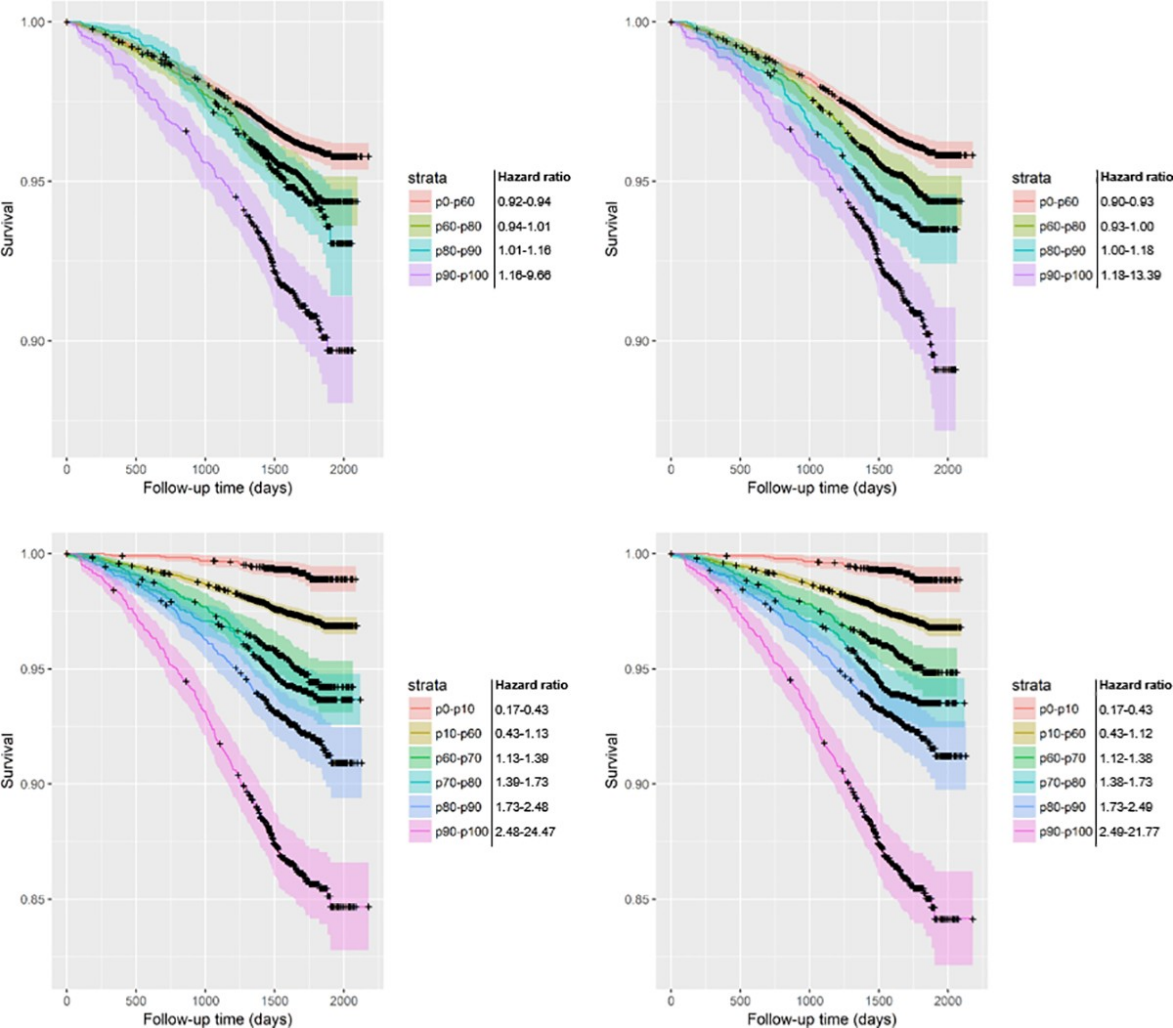
Kaplan-Meier curves of all-cause mortality survival predictions including 95% confidence intervals (resampled T0-T2 subcohort). The base models only contain the variable emphysema score; the progression models include both emphysema score and average change of emphysema score per year. Risk groups are divided by risk percentiles and their corresponding hazard ratios as displayed in the key. The resampled T0-T2 subcohort contains participants with both T0 and T2 scans resampled to the original cohort size. Upper-left: unadjusted base model; Upper-right: unadjusted two-year progression model; Lower-left: adjusted base model; Lower-right: adjusted two-year progression model.

**Fig 7:**
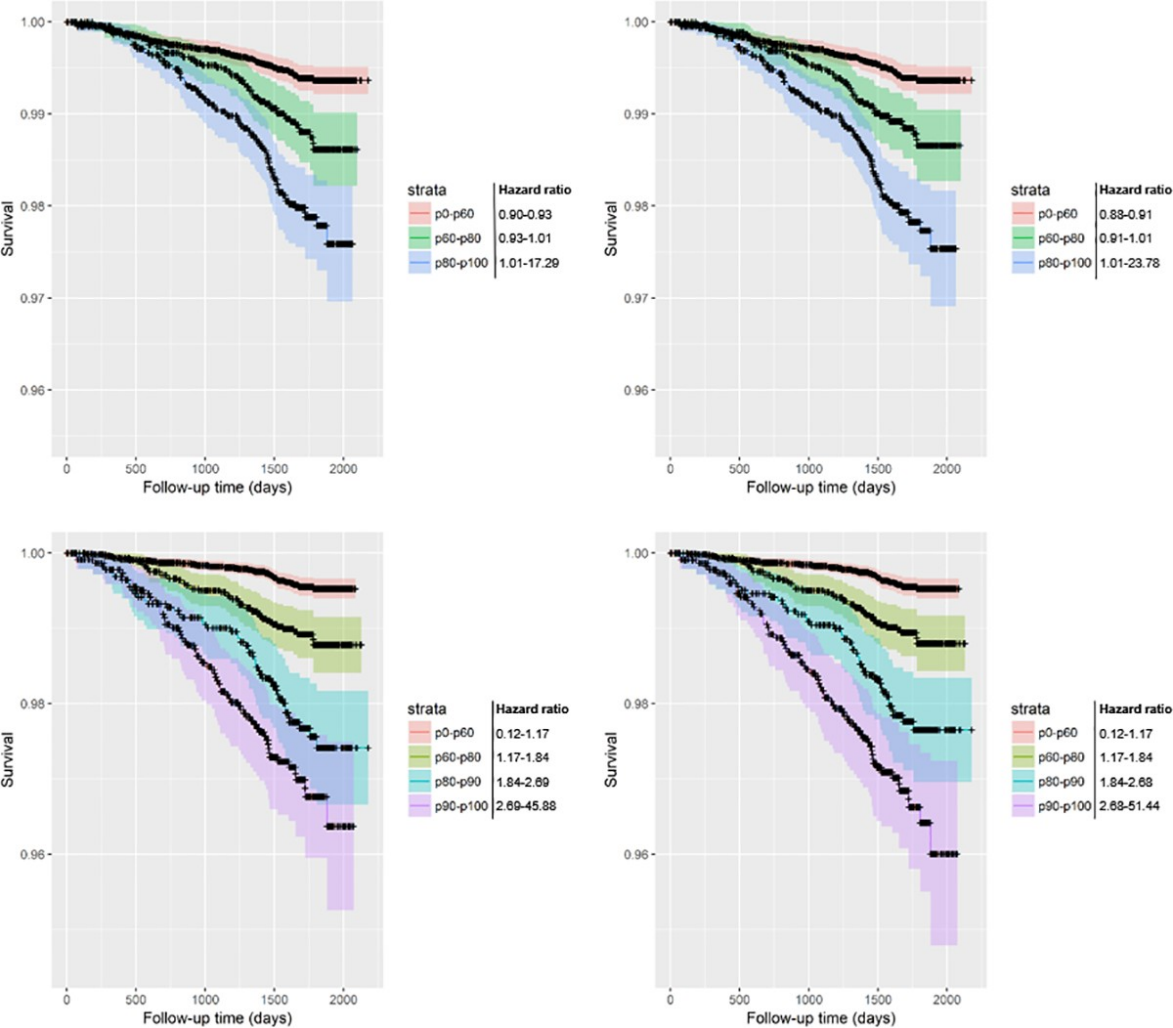
Kaplan-Meier curves of lung cancer mortality survival predictions including 95% confidence intervals (resampled T0-T2 subcohort). The base models only contain the variable emphysema score; the progression models include both emphysema score and average change of emphysema score per year. Risk groups are divided by risk percentiles and their corresponding hazard ratios as displayed in the key. The resampled T0-T2 subcohort contains participants with both T0 and T2 scans resampled to the original cohort size. Upper-left: unadjusted base model; Upper-right: unadjusted two-year progression model; Lower-left: adjusted base model; Lower-right: adjusted two-year progression model.

**Table 2:** Unadjusted and adjusted Cox regression beta coefficients and hazard ratios (resampled T0-T1 and T0-T2 subcohorts).

	All-cause mortality model			Lung cancer mortality model		
Predictor variables	β coefficient (95% CI)	HR (95% CI)	P-value	β coefficient (95% CI)	HR (95% CI)	P-value
**Resampled T0-T1 subcohort, n = 23227**						
*Unadjusted base model*						
T1 ES, per %	0.03673 (0.02981 to 0.04364)	1.037 (1.030 to 1.045)	< .001	0.04429 (0.03126 to 0.05733)	1.045 (1.032 to 1.059)	< .001
*Unadjusted one-year progression model*						
T1 ES, per %	0.05725 (0.04824 to 0.06625)	1.059 (1.049 to 1.069)	< .001	0.06347 (0.04494 to 0.08201)	1.066 (1.046 to 1.086)	< .001
T0-T1 EPR, per % per year	-0.04887 (-0.06449 to -0.03325)	0.952 (0.938 to 0.967)	< .001	-0.03965 (-0.0703 to -0.0090)	0.961 (0.932 to 0.991)	.011
*Adjusted base model*^a^						
T1 ES, per %	0.02433 (0.01588 to 0.03279)	1.025 (1.016 to 1.033)	< .001	0.03571 (0.01959 to 0.05183)	1.036 (1.020 to 1.053)	< .001
*Adjusted one-year progression model*^a^						
T1 ES, per %	0.03720 (0.02696 to 0.04745)	1.038 (1.027 to 1.049)	< .001	0.04134 (0.01987 to 0.06280)	1.042 (1.020 to 1.065)	< .001
T0-T1 EPR, per % per year	-0.03978 (-0.05921 to -0.02034)	0.961 (0.943 to 0.980)	< .001	-0.01410 (-0.05098 to 0.02277)	0.956 (0.950 to 1.023)	.454
**Resampled T0-T2 subcohort, n = 22592**						
*Unadjusted base model*						
T2 ES, per %	0.04380 (0.03601 to 0.05158)	1.045 (1.037 to 1.053)	< .001	0.05505 (0.04045 to 0.06965)	1.057 (1.041 to 1.072)	< .001
*Unadjusted two-year progression model*						
T2 ES, per %	0.06212 (0.05247 to 0.07176)	1.064 (1.054 to 1.074)	< .001	0.07356 (0.05391 to 0.09321)	1.076 (1.055 to 1.098)	< .001
T0-T2 EPR, per % per year	-0.09234 (-0.1271 to -0.05754)	0.912 (0.881 to 0.944)	< .001	-0.07532 (-0.1392 to -0.01145)	0.927 (0.870 to 0.989)	.021
*Adjusted base model*^a^						
T2 ES, per %	0.02769 (0.0185 to 0.0368)	1.028 (1.019 to 1.038)	< .001	0.03804 (0.02089 to 0.05519)	1.039 (1.021 to 1.057)	< .001
*Adjusted two-year progression model*^a^						
T2 ES, per %	0.03973 (0.02868 to 0.05079)	1.041 (1.029 to 1.052)	< .001	0.04709 (0.02401 to 0.07016)	1.048 (1.024 to 1.073)	< .001
T0-T2 EPR, per % per year	-0.07349 (-0.1161 to -0.03086)	0.929 (0.890 to 0.970)	.001	-0.03948 (-0.1112 to 0.03226)	0.961 (0.895 to 1.033)	0.281

CI, confidence interval; HR, hazard ratio; ES, emphysema score; EPR, emphysema score progression per year; T0, baseline screening round; T1, first annual follow-up screening round; T2, second annual follow-up screening round. The base models only contain the variable emphysema score; the progression models include both emphysema score and average change of emphysema score per year. The resampled T0-T1 and T0-T2 subcohorts contain participants with both T0 and T1 scans and T0 and T2 scans, respectively, resampled to the original cohort size.

^a^ Adjusted for baseline age (per year), sex (female), BMI (5 groups: <18.5, 18.5–25, 25–30, 30–35, ≥35), smoking status (active), smoking intensity (per pack-year), smoking duration (per year), previous COPD diagnosis.

**Table 3:** Adjusted Cox regression two-year progression models predicting all-cause and lung cancer mortality (resampled T0-T1 subcohort).

	All-cause mortality model			Lung cancer mortality model		
Predictor variables	β coefficient (95% CI)	HR (95% CI)	P-value	β coefficient (95% CI)	HR (95% CI)	P-value
ES, per %	0.03720 (0.02696 to 0.04745)	1.038 (1.027 to 1.049)	< .001	0.04134 (0.01987 to 0.06280)	1.042 (1.020 to 1.065)	< .001
T0 to T1 EPR, per % per year	-0.03978 (-0.05921 to -0.02034)	0.961 (0.943 to 0.980)	< .001	-0.01410 (-0.05098 to 0.02277)	0.986 (0.950 to 1.023)	.454
Age, per year	0.06221 (0.04835 to 0.07607)	1.064 (1.050 to 1.079)	< .001	0.03109 (0.00074 to 0.06143)	1.032 (1.001 to 1.063)	.045
Sex, female	-0.48305 (-0.60615 to -0.35995)	0.617 (0.545 to 0.698)	< .001	-0.30140 (-0.56166 to -0.04114)	0.740 (0.570 to 0.960)	.023
BMI < 18.5 (reference)	-	-	-	-	-	-
18.5 ≤ BMI < 25	-0.83126 (-1.25201 to -0.41051)	0.436 (0.286 to 0.663)	< .001	0.34319 (-1.06845 to 1.75482)	1.409 (0.344 to 5.782)	.634
25 ≤ BMI < 30	-1.01061 (-1.43473 to -0.58648)	0.364 (0.238 to 0.556)	< .001	0.39745 (-1.01637 to 1.81126)	1.488 (0.362 to 6.118)	.582
30 ≤ BMI < 35	-0.82620 (-1.25937 to -0.39303)	0.438 (0.284 to 0.675)	< .001	0.03087 (-1.40735 to 1.46910)	1.031 (0.245 to 4.345)	.966
BMI ≥ 35	-0.47666 (-0.92738 to -0.02594)	0.621 (0.396 to 0.974)	.038	0.38881 (-1.08211 to 1.8597)	1.475 (0.339 to 6.422)	.604
Smoking status, active	0.49238 (0.35729 to 0.62746)	1.636 (1.430 to 1.873)	< .001	0.49002 (0.19762 to 0.78243)	1.632 (1.219 to 2.187)	.001
Smoking intensity, per pack-year	0.00431 (0.00225 to 0.00637)	1.004 (1.002 to 1.006)	< .001	0.00869 (0.00466 to 0.012716)	1.009 (1.005 to 1.013)	< .001
Smoking duration, per year	0.02485 (0.01340 to 0.03629)	1.025 (1.014 to 1.037)	< .001	0.04721 (0.02153 to 0.07289)	1.048 (1.022 to 1.076)	< .001
Previous COPD diagnosis	0.61142 (0.42162 to 0.80121)	1.843 (1.524 to 2.228)	< .001	0.61740 (0.22122 to 1.01358)	1.854 (1.248 to 2.755)	.002

CI, confidence interval; HR, hazard ratio; BMI, body mass index; ES, emphysema score; EPR, ES progression per year; T0, baseline screening round; T1, second annual follow-up screening round. The progression models include both emphysema score and average change of emphysema score per year. The resampled T0- T2 subcohort contains participants with T0 and T1 scans resampled to the original cohort size.

**Table 4:** Adjusted Cox regression two-year progression models predicting all-cause and lung cancer mortality (resampled T0-T2 subcohort).

	All-cause mortality model			Lung cancer mortality model		
Predictor variables	β coefficient (95% CI)	HR (95% CI)	P-value	β coefficient (95% CI)	HR (95% CI)	P-value
ES, per %	0.03973 (0.02868 to 0.05079)	1.041 (1.029 to 1.052)	< .001	0.04709 (0.02401 to 0.07016)	1.048 (1.024 to 1.073)	< .001
T0 to T2 EPR, per % per year	-0.07349 (-0.1161 to -0.03086)	0.929 (0.890 to 0.970)	.001	-0.03948 (-1112 to 0.03226)	0.961 (0.895 to 1.033)	.281
Age, per year	0.06252 (0.04702 to 0.07802)	1.065 (1.048 to 1.081)	< .001	0.02491 (-0.0101 to 0.05996)	1.025 (0.990 to 1.062)	.164
Sex, female	-0.4854 (-0.6226 to -0.3481)	0.615 (0.537 to 0.706)	< .001	-0.2413 (-0.5372 to 0.05458)	0.786 (0.584 to 1.056)	.110
BMI < 18.5 (reference)	-	-	-	-	-	-
18.5 ≤ BMI < 25	-0.7048 (-1.188 to -0.2214)	0.494 (0.305 to 0.801)	.004	0.2409 (-1.178 to 1.660)	1.272 (0.308 to 5.261)	.739
25 ≤ BMI < 30	-0.8855 (-1.373 to -0.3981)	0.412 (0.253 to 0.672)	< .001	0.2244 (-1.200 to 1.649)	1.252 (0.301 to 5.200)	.757
30 ≤ BMI < 35	-0.6756 (-1.173 to -0.1782)	0.509 (0.309 to 0.837)	.008	-0.1929 (-1.651 to 1.265)	0.825 (0.192 to 3.544)	.795
BMI ≥ 35	-0.2912 (-0.8082 to 0.2258)	0.747 (0.446 to 1.253)	.270	0.1383 (-1.372 to 1.649)	1.148 (0.254 to 5.199)	.858
Smoking status, active	0.5259 (0.03753 to 0.6765)	1.692 (1.456 to 1.967)	< .001	0.4725 (0.1381 to 0.8070)	1.604 (1.148 to 2.241)	.006
Smoking intensity, per pack-year	0.004767 (0.002484 to 0.007050)	1.005 (1.003 to 1.007)	< .001	0.009326 (0.004671 to 0.01398)	1.009 (1.005 to 1.014)	< .001
Smoking duration, per year	0.02656 (0.01374 to 0.03938)	1.027 (1.014 to 1.040)	< .001	0.04925 (0.01963 to 0.07884)	1.050 (1.020 to 1.082)	.001
Previous COPD diagnosis	0.7316 (0.5266 to 0.9365)	2.078 (1.693 to 2.551)	< .001	0.8233 (0.3944 to 1.252)	2.278 (1.484 to 3.498)	< .001

CI, confidence interval; HR, hazard ratio; BMI, body mass index; ES, emphysema score; EPR, ES progression per year; T0, baseline screening round; T2, second annual follow-up screening round. The progression models include both emphysema score and average change of emphysema score per year. The resampled T0- T2 subcohort contains participants with T0 and T2 scans resampled to the original cohort size.

Time-dependent AUCs and NRIs of the base and progression models are displayed in Tables 5 and 6. For both outcomes and across most time points, the resulting AUCs and NRIs indicated that the progression models had significantly higher accuracies than the base models when not adjusted for patient characteristics. When adjusted for patient characteristics, progression models were not statistically superior to the base models at most time points.

**Table 5:** Time-dependent receiver operating characteristic area under the curve comparing the emphysema score base models to the progression models predicting all-cause mortality and lung cancer mortality (resampled T0-T1 and T0-T2 subcohorts).

**Resampled T0-T1 subcohort**				
	**Unadjusted models**		**Adjusted models**	
**Years after T1**	**Base model AUC (95% CI)**	**One-year progression model AUC (95% CI)**^**a**^	**Base model AUC (95% CI)**	**One-year progression model AUC (95% CI)**^**a**^
**All-cause mortality prediction**				
**1**	0.537 (0.535 to 0.539)	0.542 (0.540 to 0.544)	0.698 (0.671 to 0.725)	0.707 (0.677 to 0.738)
**2**	0.530 (0.529 to 0.531)	0.535 (0.533 to 0.536)	0.678 (0.676 to 0.679)	0.681 (0.680 to 0.683)
**3**	0.546 (0.545 to 0.547)	0.554 (0.553 to 0.555)	0.700 (0.699 to 0.701)	0.702 (0.701 to 0.702)
**4**	0.552 (0.551 to 0.552)	0.560 (0.560 to 0.561)	0.709 (0.708 to 0.710)	0.710 (0.709 to 0.711)
**5**	0.559 (0.559 to 0.560)	0.566 (0.565 to 0.566)	0.710 (0.709 to 0.711)	0.711 (0.710 to 0.711)
**6**	0.564 (0.564 to 0.565)	0.569 (0.568 to 0.569)	0.704 (0.703 to 0.704)	0.705 (0.704 to 0.705)
**Lung cancer mortality prediction**				
**1**	0.631 (0.625 to 0.638)	0.681 (0.675 to 0.687)	0.777 (0.772 to 0.781)	0.777 (0.772 to 0.782)
**2**	0.576 (0.573 to 0.578)	0.579 (0.577 to 0.582)	0.719 (0.717 to 0.721)	0.721 (0.719 to 0.723)
**3**	0.579 (0.577 to 0.581)	0.591 (0.590 to 0.593)	0.705 (0.703 to 0.706)	0.706 (0.705 to 0.708)
**4**	0.613 (0.611 to 0.614)	0.626 (0.624 to 0.627)	0.730 (0.728 to 0.731)	0.731 (0.730 to 0.733)
**5**	0.632 (0.631 to 0.633)	0.641 (0.640 to 0.642)	0.730 (0.729 to 0.731)	0.731 (0.730 to 0.732)
**6**	0.638 (0.637 to 0.639)	0.643 (0.642 to 0.644)	0.724 (0.723 to 0.725)	0.725 (0.725 to 0.726
**Resampled T0-T2 subcohort**				
	**Unadjusted models**		**Adjusted models**	
**Years after T2**	**Base model AUC (95% CI)**	**Two-year progression model AUC (95% CI)^a^**	**Base model AUC (95% CI)**	**Two-year progression model AUC (95% CI)^a^**
**All-cause mortality prediction**				
**1**	0.522 (0.520 to 0.524)	0.539 (0.537 to 0.540)	0.680 (0.678 to 0.681)	0.681 (0.679 to 0.682)
**2**	0.547 (0.546 to 0.548)	0.568 (0.567 to 0.569)	0.709 (0.708 to 0.710)	0.711 (0.710 to 0.711)
**3**	0.563 (0.562 to 0.563)	0.576 (0.575 to 0.576)	0.713 (0.712 to 0.714)	0.714 (0.713 to 0.714)
**4**	0.569 (0.568 to 0.570)	0.578 (0.577 to 0.578)	0.714 (0.713 to 0.714)	0.714 (.714 to 0.715)
**5**	0.576 (0.575 to 0.576)	0.582 (0.582 to 0.583)	0.709 (0.709 to 0.710)	0.710 (0.709 to 0.710)
**Lung cancer mortality prediction**				
**1**	0.548 (0.544 to 0.553)	0.548 (0.544 to 0.552)	0.688 (0.684 to 0.692)	0.692 (0.688 to 0.695)
**2**	0.595 (0.593 to 0.597)	0.605 (0.603 to 0.607)	0.736 (0.734 to 0.738)	0.739 (0.737 to 0.741)
**3**	0.647 (0.645 to 0.648)	0.651 (0.650 to 0.653)	0.754 (0.753 to 0.756)	0.757 (0.755 to 0.758)
**4**	0.660 (0.658 to 0.661)	0.664 (0.663 to 0.665)	0.753 (0.752 to 0.754)	0.755 (0.753 to 0.756)
**5**	0.666 (0.665 to 0.667)	0.669 (0.667 to 0.670)	0.741 (0.740 to 0.742)	0.743 (0.742 to 0.743)

AUC, receiver operating characteristic area under the curve; CI, confidence interval; T0, baseline screening round; T1, first annual follow-up screening round; T2, second annual follow-up screening round. The base models only contain the variable emphysema score; the progression models include both emphysema score and average change of emphysema score per year. The resampled T0-T1 and T0-T2 subcohorts contain participants with both T0 and T1 scans and T0 and T2 scans, respectively, resampled to the original cohort size.

^a^ Adjusted for baseline age (per year), sex (female), BMI (5 groups: <18.5, 18.5–25, 25–30, 30–35, ≥35), smoking status (active), smoking intensity (per pack-year), smoking duration (per year), previous COPD diagnosis.

**Table 6:** Time-dependent average continuous net reclassification improvement comparing the emphysema score base models to the progression models (resampled T0-T1 and T0-T2 subcohorts).

	All-cause mortality models		Lung cancer mortality models	
**Base model versus one-year progression model**				
**Years after T1**	**Unadjusted models NRI (95% CI)**	**Adjusted models NRI (95% CI)^a^**	**Unadjusted models NRI (95% CI)**	**Adjusted models NRI (95% CI)^a^**
**1**	10.7% (2.6 to 18.9%)	1.2% (-7.8 to 10.2%)	37.9% (7.3 to 66.7%)	-6.3% (-30.9 to 33.1%)
**2**	9.5% (3.8 to 14.2%)	1.7% (-5.4 to 7.6%)	12.7% (0.7 to 24.8%)	-8.9% (-20.4 to 17.0%)
**3**	8.8% (4.9 to 12.2%)	1.1% (-4.1 to 5.7%)	13.5% (3.7 to 21.3%)	-4.4% (-13.8 to 13.8%)
**4**	7.9% (4.8 to 10.9%)	-0.8% (-5.5 to 3.2%)	16.7% (8.8 to 24.3%)	-1.7% (-11.2 to 12.1%)
**5**	8.6% (5.6 to 10.8%)	1.1% (-3.9 to 4.8%)	16.4% (10.1 to 22.0%)	-1.7% (-10.2 to 10.5%)
**6**	10.2% (6.8 to 12.8%)	3.9% (-0.8 to 8.0%)	17.0% (11.4 to 23.1%)	2.2% (-7.5 to 10.1%)
**Base model versus two-year progression models**				
**Years after T2**	**Unadjusted models NRI (95% CI)**	**Adjusted models NRI (95% CI)^a^**	**Unadjusted models NRI (95% CI)**	**Adjusted models NRI (95% CI)^a^**
**1**	7.5% (-0.4 to 14.4%)	0.6% (-6.6 to 11.7%)	02.3% (-11.2 to 21.1%)	-6.7% (-23.9 to 19.1%)
**2**	12.0% (6.7 to 17.4%)	1.1% (-3.7 to 8.6%)	13.2% (3.3 to 22.3%)	-0.7% (-10.9 to 15.6%)
**3**	9.4% (5.6 to 13.4%)	-1.0% (-5.8 to 4.8%)	13.9% (6.7 to 22.0%)	-0.2% (-8.7 to 13.9%)
**4**	9.7% (6.2 to 12.3%)	-1.1% (-5.8 to 3.2%	14.5% (8.8 to 20.9%)	-1.4% (-8.3 to 11.6%)
**5**	12.3% (9.4 to 15.9%)	5.3% (0.8 to 9.9%)	17.6% (12.2 to 24.4%)	6.4% (-5.6 to 16.3%)

CI, confidence interval; NRI, average continuous net reclassification improvement; T0, baseline screening round; T1, first annual follow-up screening round; T2, second annual follow-up screening round. NRI is a model comparison measure that produces a number between -1 to 1; here, a number above 0 signifies that the progression model is superior to the base progression model, more so as the NRI approaches 1. The base models only contain the variable emphysema score; the progression models include both emphysema score and average change of emphysema score per year. The resampled T0-T1 and T0-T2 subcohorts contain participants with both T0 and T1 scans and T0 and T2 scans, respectively, resampled to the original cohort size.

^a^ Adjusted for baseline age (per year), sex (female), BMI (5 groups: <18.5, 18.5–25, 25–30, 30–35, ≥35), smoking status (active), smoking intensity (per pack-year), smoking duration (per year), previous COPD diagnosis.

Measures of clinical outcomes at several hazard ratio cut-off points (based on percentiles) are displayed in Table 7. Without information on patient characteristics, 80% of the participants had an estimated hazard ratio above 1.00 using both the base and one-year progression models. At this cut-off point, 29.1% (95% confidence interval: 26.7 to 31.7%) of all the deaths within six years were correctly predicted using solely T1 ES; per death, there were 11.3 (10.1 to 12.6) surviving “overdiagnosed” participants. If only 2% of those with the highest risk scores were selected, 4.7% (3.6 to 6.0%) of all deaths would be observed; however, the ratio of non-deceased to deceased would also decrease to 6.6 (4.8 to 9.4). With the addition of EPR, this ratio decreases to 5.4 (4.0 to 7.5); the ratios for the adjusted base and the adjusted one-year progression models are 3.5 (2.6 to 4.6) and 3.3 (2.5 to 4.4), respectively.

**Table 7:** Models’ clinical outcomes predicting 6-year all-cause mortality at several hazard cut-off points (T0-T1 subcohort).

Model	Cut-off percentile (HR)	Deaths within 6 years	Survivors after 6 years	Sensitivity (%) (CI)	Specificity (%) (CI)	Positive predictive value (%) (CI)	Negative predictive value (%) (CI)	Number of survivors per death (CI)
Follow all participants	0 (NA)	1302	21925	100.0 (99.6 to 100.0)	0.0 (0.0 to 0.0)	5.6 (5.6 to 5.6)	NA	16.8 (16.8 to 16.9)
Unadjusted base model	80 (1.00)	379	4269	29.1 (26.7 to 31.7)	80.5 (80.0 to 81.0)	8.2 (7.3 to 9.0)	95.0 (94.8 to 95.2)	11.3 (10.1 to 12.6)
	90 (1.13)	220	2105	16.9 (14.9 to 19.1)	90.4 (90.0 to 90.8)	9.5 (8.1 to 11.0)	94.8 (94.7 to 95.0)	9.6 (8.1 to 11.3)
	95 (1.37)	123	1039	9.4 (7.9 to 11.2)	95.3 (95.0 to 95.5)	10.6 (8.6 to 12.9)	94.7 (94.6 to 94.8)	8.4 (6.8 to 10.7)
	98 (1.82)	61	404	4.7 (3.6 to 6.0)	98.2 (98.0 to 98.3)	13.1 (9.7 to 17.3)	94.5 (94.5 to 94.6)	6.6 (4.8 to 9.4)
Unadjusted one-year progression model	80 (1.00)	388	4258	29.8 (27.3 to 32.4)	80.6 (90.0 to 81.1)	8.4 (7.5 to 9.2)	95.1 (94.9 to 95.3)	11.0 (9.8 to 12.3)
	90 (1.16)	226	2104	17.4 (15.4 to 19.6)	90.4 (90.0 to 90.8)	9.7 (8.4 to 11.2)	94.9 (94.7 to 95.0)	9.3 (7.9 to 10.9)
	95 (1.49)	138	1024	10.6 (9.0 to 12.4)	95.3 (95.0 to 95.6)	11.9 (9.7 to 14.3)	94.7 (94.6 to 94.8)	7.4 (6.0 to 9.4)
	98 (2.16)	74	398	5.7 (4.5 to 7.1)	98.2 (98.0 to 98.3)	15.7 (11.8 to 19.9)	94.6 (94.5 to 94.7)	5.4 (4.0 to 7.5)
Adjusted base model	80 (1.71)	579	4071	44.5 (41.8 to 47.2)	81.4 (80.9 to 81.9)	12.5 (11.5 to 13.4)	96.1 (95.9 to 96.3)	7.0 (6.5 to 7.7)
	90 (2.43)	361	1962	27.7 (25.3 to 30.3)	91.1 (90.7 to 91.4)	15.5 (13.9 to 17.3)	95.5 (95.3 to 95.7)	5.4 (4.8 to 6.2)
	95 (3.15)	219	945	16.8 (14.9 to 19.0)	95.7 (95.4 to 95.9)	18.8 (16.1 to 21.6)	95.1 (95.0 to 95.2)	4.3 (3.6 to 5.2)
	98 (4.62)	104	361	8.0 (6.6 to 9.6)	98.4 (98.0 to 98.3)	22.4 (17.9 to 27.5)	94.7 (94.7 to 94.8)	3.5 (2.6 to 4.6)
Adjusted one-year progression model	80 (1.70)	578	4069	44.4 (41.7 to 47.1)	81.4 (80.9 to 81.9)	12.4 (11.5 to 13.4)	96.1 (95.9 to 96.3)	7.0 (6.5 to 7.7)
	90 (2.42)	368	1959	28.3 (25.8 to 30.8)	91.1 (90.7 to 91.4)	15.8 (14.1 to 17.5)	95.5 (95.4 to 95.7)	5.3 (4.7 to 6.1)
	95 (3.21)	226	936	17.4 (15.4 to 19.6)	95.7 (95.4 to 96.0)	19.4 (16.6 to 22.5)	95.1 (95.0 to 95.3)	4.1 (3.4 to 5.0)
	98 (4.72)	108	358	8.3 (6.9 to 10.0)	98.4 (98.2 to 98.5)	23.2 (18.5 to 28.4)	94.8 (94.7 to 94.9)	3.3 (2.5 to 4.4)

CI, 95% confidence interval; HR, hazard ratio; NA, not applicable. In the hypothetical situation where screened participants are selected for an additional six years of follow-up based on our models’ estimated hazard ratios, these would be the clinical outcomes of the selected participants at several risk cut-off points.

### One- versus two-year progression models

Two ES progression models were developed from the resampled T0-T1-T2 subcohort: the one- and two-year EPR calculated from T1 and T2 scans and T0 and T2 scans, respectively. With or without adjustment factors, the absolute value of the two-year EPR beta coefficients (unadjusted: -0.08460 [-0.12219 to -0.04702]; adjusted: -0.06288 [-0.10910 to -0.01665]) were more than four times larger than those of one-year EPR (unadjusted: -0.02025 [-0.03142 to -0.00908]; adjusted: -0.01323 [-0.02776 to 0.00130]) when predicting all-cause mortality (Table 8). At most time points, the AUCs of the unadjusted progression models indicated a small gain in accuracy when using the two-year progression model compared to the one-year progression model (Table 9); the difference in AUCs ranged from 0.002 to 0.013. The differences in beta coefficients for predicting lung cancer mortality were smaller, had a larger variance, and did not lead to differences in model performance. No performance differences were seen among the adjusted models’ AUCs. Adjusted or not, the time-dependent NRI comparisons did not show a statistically significant difference between the progression models (Table 10).

**Table 8:** Unadjusted and adjusted Cox regression beta coefficients and hazard ratios (resampled T0-T1-T2 subcohort).

	All-cause mortality model			Lung cancer mortality model		
Predictor variables	β coefficient (95% CI)	HR (95% CI)	P-value	β coefficient (95% CI)	HR (95% CI)	P-value
Resampled T0-T1-T2 subcohort, n = 21679						
*Unadjusted base model*						
T2 ES, per %	0.04592 (0.03772 to 0.05413)	1.047 (1.038 to 1.056)	< .001	0.05457 (0.03849 to 0.07066)	1.056 (1.039 to 1.073)	< .001
*Unadjusted one-year progression model*						
T2 ES, per %	0.05273 (0.04372 to 0.06175)	1.054 (1.045 to 1.064)	< .001	0.06316 (0.04503 to 0.08129)	1.065 (1.046 to 1.085)	< .001
T1-T2 EPR, per % per year	-0.02025 (-0.03142 to -0.00908)	0.980 (0.969 to 0.991)	< .001	-0.02221 (-0.04456 to 0.00013)	0.978 (0.956 to 1.000)	.051
*Unadjusted two-year progression model*						
T2 ES, per %	0.06137 (0.05138 to 0.07135)	1.063 (1.053 to 1.074)	< .001	0.06737 (0.04590 to 0.08884)	1.070 (1.047 to 1.093)	< .001
T0-T2 EPR, per % per year	-0.08460 (-0.12219 to -0.04702)	0.919 (0.885 to 0.954)	< .001	-0.05486 (-0.12486 to 0.01514)	0.947 (0.883 to 1.015)	.125
*Adjusted base model*^*a*^						
T2 ES, per %	0.03077 (0.02115 to 0.04038)	1.031 (1.021 to 1.041)	< .001	0.04011 (0.02114 to 0.05908)	1.041 (1.021 to 1.061)	< .001
*Adjusted one-year progression model*^*a*^						
T2 ES, per %	0.03401 (0.02368 to 0.04433)	1.035 (1.024 to 1.045)	< .001	0.04507 (0.02396 to 0.06619)	1.046 (1.024 to 1.068)	< .001
T1-T2 EPR, per % per year	-0.01323 (-0.02776 to 0.00130)	0.987 (0.973 to 1.001)	.074	-0.01652 (-0.04601 to 0.01296)	0.984 (0.955 to 1.013)	.272
*Adjusted two-year progression model*^*a*^						
T2 ES, per %	0.03996 (0.02860 to 0.05133)	1.041 (1.029 to 1.053)	< .001	0.04277 (0.01788 to 0.06765)	1.044 (1.018 to 1.070)	< .001
T0-T2 EPR, per % per year	-0.06288 (-0.10910 to -0.01665)	0.939 (0.897 to 0.984)	.008	-0.01252 (-0.09019 to 0.06514)	0.988 (0.914 to 1.067)	.752

CI, confidence interval; HR, hazard ratio; ES, emphysema score; EPR, ES progression per year; T0, baseline screening round; T1, first annual follow-up screening round; T2, second annual follow-up screening round. The base models only contain the variable emphysema score; the progression models include both emphysema score and average change of emphysema score per year. The resampled T0-T1-T2 subcohort contains participants with all T0, T1, and T2 scans resampled to the original cohort size.

^a^ Adjusted for baseline age (per year), sex (female), BMI (5 groups: <18.5, 18.5–25, 25–30, 30–35, ≥35), smoking status (active), smoking intensity (per pack-year), smoking duration (per year), previous COPD diagnosis.

**Table 9:** Time-dependent receiver operating characteristic area under the curve comparing the base models against the progression models (resampled T0-T1-T2 subcohort).

**All-cause mortality prediction**						
	**Unadjusted models**			**Adjusted models**		
**Years after T2**	**Base model AUC (95% CI)**	**One-year progression model AUC (95% CI)**	**Two-year progression model AUC (95% CI)**	**Base model AUC (95% CI)**	**One-year progression model AUC (95% CI)**	**Two-year progression model AUC (95% CI)**
**1**	0.520 (0.518 to 0.522)	0.524 (0.523 to 0.526)	0.535 (0.534 to 0.537)	0.675 (0.674 to 0.676)	0.674 (0.673 to 0.676)	0.676 (0.674 to 0.677)
**2**	0.545 (0.544 to 0.546)	0.550 (0.549 to 0.551)	0.563 (0.562 to 0.565)	0.707 (0.707 to 0.708)	0.707 (0.707 to 0.708)	0.708 (0.707 to 0.709)
**3**	0.565 (0.564 to 0.566)	0.568 (0.567 to 0.569)	0.577 (0.576 to 0.578)	0.713 (0.712 to 0.713)	0.713 (0.712 to 0.714)	0.713 (0.712 to 0.714)
**4**	0.569 (0.568 to 0.570)	0.570 (0.570 to 0.571)	0.576 (0.575 to 0.576)	0.712 (0.711 to 0.713)	0.712 (0.712 to 0.713)	0.712 (0.712 to 0.713)
**5**	0.576 (0.575 to 0.576)	0.577 (0.577 to 0.578)	0.581 (0.580 to 0.582)	0.708 (0.707 to 0.708)	0.708 (0.708 to 0.709)	0.708 (0.708 to 0.709)
**Lung cancer mortality prediction**						
	**Unadjusted models**			**Adjusted models**		
**Years after T2**	**Base model AUC (95% CI)**	**One-year progression model AUC (95% CI)**	**Two-year progression model AUC (95% CI)**	**Base model AUC (95% CI)**	**One-year progression model AUC (95% CI)**	**Two-year progression model AUC (95% CI)**
**1**	0.512 (0.508 to 0.547)	0.513 (0.508 to 0.517)	0.510 (0.506 to 0.514)	0.664 (0.661 to 0.668)	0.665 (0.661 to 0.668)	0.666 (0.663 to 0.670)
**2**	0.573 (0.571 to 0.575)	0.572 (0.570 to 0.575)	0.577 (0.575 to 0.580)	0.735 (0.733 to 0.737)	0.735 (0.733 to 0.737)	0.736 (0.734 to 0.737)
**3**	0.643 (0.641 to 0.645)	0.638 (0.636 to 0.340)	0.645 (0.643 to 0.646)	0.755 (0.754 to 0.757)	0.755 (0.753 to 0.756)	0.756 (0.754 to 0.757)
**4**	0.655 (0.654 to 0.656)	0.652 (0.651 to 0.653)	0.657 (0.656 to 0.658)	0.751 (0.750 o 0.752)	0.751 (0.750 to 0.752)	0.752 (0.751 to 0753)
**5**	0.662 (0.660 to 0.663)	0.660 (0.659 to 0.661)	0.662 (0.661 to 0.664)	0.738 (0.737 to 0.739)	0.739 (0.738 to 0.740)	0.739 (0.738 to 0.340)

AUC, receiver operating characteristic area under the curve; CI, confidence interval; T0, baseline screening round; T1, first annual follow-up screening round; T2, second annual follow-up screening round. The base models only contain the variable emphysema score; the progression models include both emphysema score and average change of emphysema score per year. The resampled T0-T1-T2 subcohort contains participants with all T0, T1, and T2 scans resampled to the original cohort size.

**Table 10:** Time-dependent average continuous net reclassification improvement comparing the one-year to the two-year emphysema score progression models (resampled T0-T1-T2 subcohort).

	All-cause mortality models		Lung cancer mortality models	
Years after T2	Unadjusted models NRI (95% CI)	Adjusted models NRI (95% CI)^a^	Unadjusted models NRI (95% CI)	Adjusted models NRI (95% CI)^a^
**1**	-1.1% (-8.4 to 9.4%)	3.1% (-10.5 to 11.4%)	-10.9% (-29.5 to 9.7%)	-0.5% (-26.4 to 27.8%)
**2**	5.2% (-2.2 to 12.1%)	0.1% (-7.8 to 6.4%)	-1.7% (-14.9 to 18.3%)	-7.4% (-22.8 to 14.9%)
**3**	6.6% (-3.9 to 11.3%)	-2.5% (-8.5 to 2.6%)	-1.3% (-11.5 to 24.2%)	-4.8% (-16.6 to 12.4%)
**4**	7.4% (-4.2 to 11.3%)	-3.0% (-8.0 to 2.4%)	-5.8% (-13.4 to 21.0%)	-6.6% (-15.6 to 10.7%)
**5**	8.7% (-3.9 to 13.2%)	0.8% (-5.2 to 6.6%)	-8.6% (-15.7 to 20.4%)	-11.5% (-18.0 to 8.3%)

CI, confidence interval; NRI, average continuous net reclassification improvement; T0, baseline screening round; T1, first annual follow-up screening round; T2, second annual follow-up screening round. NRI is a model comparison measure that produces a number between -1 to 1; here, a number above 0 signifies that the two-year progression model is superior to the one-year progression model, more so as the NRI approaches 1. The progression models include both emphysema score and average change of emphysema score per year. The resampled T0-T1-T2 subcohort contains participants with all T0, T1, and T2 scans resampled to the original cohort size.

^a^ Adjusted for baseline age (per year), sex (female), BMI (5 groups: <18.5, 18.5–25, 25–30, 30–35, ≥35), smoking status (active), smoking intensity (per pack-year), smoking duration (per year), previous COPD diagnosis.

## Discussion

To our best knowledge, no other study has yet attempted to measure pulmonary emphysema progression in CT scan using quantitative methods for potential use as a measure of disease progression over time. Our study demonstrated that when a prior chest CT scan is available, calculating the EPR in addition to the latest ES showed a very minor improvement to the all-cause and lung cancer mortality prediction in a lung cancer screening population. On the other hand, when baseline patient information was available and included in the model–more specifically age, sex, weight, height, smoking history, and disease history–the value of EPR is negated completely. These results are also reflected in Table 7, which shows the predicted clinical outcomes when the population is separated into high versus low risk groups.

Though not an objective of the study, a positive result was that ES remained a highly significant predictor in all adjusted models developed (p < .001); this had not yet been demonstrated in the previous study by Gallardo-Estrella et al. [9]. ES should therefore continue to be considered for inclusion in future multivariable risk prediction models when available.

Among most progression models, it was observed that the absolute value of the models’ two-year EPR beta coefficients were considerably larger than that of one-year EPR. A theory is that that calculating EPR using a larger time gap between two images would reduce the within-participant random measurement errors relative to the actual change in ES. We therefore hypothesized that two-year EPR is a superior mortality biomarker than one-year EPR and developed both models in a common data set where each participant had images from all screening rounds. Indeed, in the resampled T0-T1-T2 subcohort, we found a statistically significant higher AUC for the unadjusted two-year progression model than that of one-year; on the other hand, the magnitude was very small and again completely negated when adjusted for patient characteristics.

It is worth noting that most participants do not have an EPR large enough to have any influence on their risk score. Using a non-parametric test, there was no statistically significant difference in EPR between the deceased and non-deceased groups (Table 1). However, even among heavy smokers (at least 30 pack years, and no more than 15 years since quit among former smokers [8]), only 6.3% (359/5740) had the diagnosis COPD at baseline. The added value may therefore only apply for the small proportion with more severe emphysema. Careful consideration of the Kaplan-Meier curves reveals that the biggest change generally takes place in the highest risk group (p90-p100) (Figs 4–7). In most graphs, the five- or six-year survival probability of the p90-p100 risk group increases by about 1% and the p80-p90 group decreases by about 1% accompanied by a narrowing of confidence intervals for better separation from the p60-p80 group. Graphs of adjusted models–which could better distinguish between lower risk strata–additionally showed an improved separation between the p60-p70 and p70-p80 groups. In other words, the seemingly negligible benefit of EPR may in part be due to the small number of people that it applies to.

Another discussion point is that emphysema is currently considered permanent and irreversible, so a moderate decrease in ES which is not explainable by random within-participant measurement errors should not be possible. Regardless, the EPR beta coefficients among all progression models were negative, meaning that a decrease in ES since the last scan increases the mortality risk. This is a paradoxical result which has been observed in previous studies [18–23]. The current explanation for this is that active smokers’ lungs are in a constant state of inflammation causing a diffuse increase in density of the lung parenchyma; smoking cessation leads to the clearance of the inflammatory component resulting in a general decrease of lung density and revealing areas of decreased lung density below the threshold of being counted as emphysema, hereby increasing the ES. In a study cohort where about half of the participants were diagnosed with COPD, Grydeland and colleagues [18] reported non-normalized ES medians of 9.72 and 4.86 among former and current smokers, respectively. In our study, 40 out of the 4420 participants (0.9%) in our T0-T1-T2 subcohort had a T0 to T1 EPR less than -5 and 103 (2.3%) met these criteria from T1 to T2. On the other side of the spectrum, the numbers with an EPR greater than 5 were 135 (3.1%) and 93 (2.1%), respectively.

In our study, adjusting for whether the participant was an active smoker or not did not change the sign; this information was taken from a baseline questionnaire, hence it is not known whether the participants’ smoking behavior had changed. It may therefore be conjectured that former smokers relapsed after the screening program failed to diagnose them with lung cancer [24]. Scan normalization does not change the overall scan density and can therefore not automatically adjust for changes in inflammation over time. To solve this issue, different density cut-off points may be used depending on the time since smoking abstinence. Another solution may be to adjust for the mean lung density, which would not require information on smoking behavior.

This study has other limitations. Due to the very small spread, it is still not clear what kind of relationship EPR has with mortality risk; experimenting with various transformation methods did not appear to improve the linearity nor the model accuracy (results not included). Our inclusion criteria required participants to have survived one or two years following the baseline scan, which introduced selection bias.

The value of the current study is that it provides a thorough description and reasoning of the unexpected negative findings, which may contribute to ensuing investigations. A future study on validating EPR should either utilize a data set including patient characteristics reported at each scan time point or include another CT measure to adjust for differences in overall lung density.

## Conclusions

It is expected that the prognosis accuracy of a disease should improve considerably when the rate of disease progression is known, which can only be recorded when measurements are made at multiple time points. This was not the case in our study, where we have shown that simply calculating the change in normalized emphysema score between two CT images does not clinically improve the prediction of all-cause and lung cancer mortality. We surmised that recent smoking behavior has a crucial impact on the emphysema score: Though the normalization method remains useful for comparing CT images created using different protocols and algorithms, it does not adjust for changes in the overall lung density which, for each individual, is prone to change over time. It is expected that a future study utilizing follow-up clinical information or adjusting for the mean lung density is necessary before emphysema score progression rate may be clinically useful as a quantitative CT biomarker.

## Supporting information

S1 Dataset Dataset of emphysema scores and emphysema score progression rates. (CSV)

## Abbreviations

AUC: area under the receiver operating characteristic curve
BMI: body mass index
CT: computed tomography
NLST: National Lung Screening Trial
ES: emphysema score
NRI: average continuous net reclassification improvement
EPR: emphysema score progression rate
